# EPH/Ephrin-Targeting Treatment in Breast Cancer: A New Chapter in Breast Cancer Therapy

**DOI:** 10.3390/ijms232315275

**Published:** 2022-12-03

**Authors:** Iason Psilopatis, Eleni Souferi-Chronopoulou, Kleio Vrettou, Constantinos Troungos, Stamatios Theocharis

**Affiliations:** 1 First Department of Pathology, Medical School, National and Kapodistrian University of Athens, 75 Mikras Asias Street, Building 10, Goudi, 11527 Athens, Greece; 2 Department of Gynecology, Charité-Universitätsmedizin Berlin, Corporate Member of Freie Universität Berlin and Humboldt-Universität zu Berlin, Augustenburger Platz 1, 13353 Berlin, Germany; 3 Department of Biological Chemistry, Medical School, National and Kapodistrian University of Athens, 75 Mikras Asias Street, Building 10, Goudi, 11527 Athens, Greece

**Keywords:** ephrin receptor, EPH, ephrin, breast cancer, treatment

## Abstract

Breast cancer (BC) is the most common malignant tumor in women. Erythropoietin-producing hepatocellular receptors (EPHs), receptor tyrosine kinases binding the membrane-bound proteins ephrins, are differentially expressed in BC, and correlate with carcinogenesis and tumor progression. With a view to examining available therapeutics targeting the EPH/ephrin system in BC, a literature review was conducted, using the MEDLINE, LIVIVO, and Google Scholar databases. EPHA2 is the most studied EPH/ephrin target in BC treatment. The targeting of EPHA2, EPHA10, EPHB4, ephrin-A2, ephrin-A4, as well as ephrin-B2 in BC cells or xenograft models is associated with apoptosis induction, tumor regression, anticancer immune response activation, and impaired cell motility. In conclusion, EPHs/ephrins seem to represent promising future treatment targets in BC.

## 1. Introduction

Breast cancer (BC) represents the most common malignant tumor in women in the United States [1]. According to the American Cancer Society, about 287,850 new cases of invasive BC will be diagnosed and about 43,250 women will die from BC in the United States in 2022 [1]. Patients with BC usually present with a new, palpable, nontender, and firm mass, with poorly defined edges. In some cases, women may also describe pain, retractions, dimpling, peau d’orange, nipple inversion, or even blood-tinged discharge [2]. Diagnostic evaluation of BC includes, in addition to a clinical breast examination, breast ultrasound or mammography, depending on the patient’s age, as well as breast Magnetic Resonance Imaging (MRI). A definite diagnosis always requires a biopsy of the tumor mass [3]. For women with early-stage resectable BC, breast-conserving surgery, along with radiotherapy and adjuvant hormone (for hormone receptor-positive BC) or targeted (for human epidermal growth factor receptor 2 (HER2)- positive BC) therapy, represent the first-line of therapy. Patients with more advanced stage BC may be also treated with a mastectomy and (neo-) adjuvant chemotherapy [4].

Erythropoietin-producing hepatocellular receptors (EPHs) build the largest subfamily of receptor tyrosine kinases, binding the so-called membrane-bound protein ephrins [5]. Based on their structural homology, the two subfamilies of EPHs, EPHAs, and EPHBs, preferentially bind ephrin-A and ephrin-B ligands, respectively [6,7]. In particular, nine EPHA receptors (EPHA1-8, 10) bind five ephrin-A ligands (ephrin-A1-5), while five EPHB receptors (EPHB1-4, 6) interact with three ephrin-B ligands (ephrin-B1-3) in humans [8]. In terms of receptor–ligand interaction, ephrin-As interact with EPHAs via a glycosylphosphatidylinositol anchor on plasma membranes, whereas ephrin-Bs tether EPHBs to the membrane by a transmembrane domain [9]. EPHs, together with their ephrin ligands, are widely expressed in numerous cell types, and have been described to play an important role in cell migration, cell-to-cell or cell-matrix interaction, as well as (lymph-) angiogenesis [10,11]. Due to their implication in all these physiological functions, the EPH/ephrin system is provenly involved in tumor development and progression, as well [12,13,14,15,16].

Given its high incidence and clinical relevance, multiple study groups have investigated the role of the EPH/ephrin system in BC [17,18,19]. Recently, Nikas et al. meticulously summarized the results of preclinical studies incorporating BC cell lines/animal models, as well as studies showing the clinical significance of aberrant EPH/ephrin expression patterns in human material, and published their mini-review on EPHs/ephrins, with a focus on BC heterogeneity [20]. EPHA2, EPHB4, and EPHB6 represent the most extensively studied members of the EPH/ephrin system in BC, while the expression of EPHA2-7, EPHA10, EPHB2, EPHB4, EPHB6, ephrin-A1, ephrin-A3, and ephrin-B1 in BC samples clinically correlates with the immunohistochemistry (IHC)-based groups, grading, staging, and survival. In particular, high levels of EPHA2 correlate with adverse prognosis in triple-negative (TNBC), hormone receptor-positive, as well as HER2-positive BC, with EPHA2 participating in the resistance mechanisms against both antihormonal (antiestrogens) and targeted (trastuzumab) therapy [20]. Similarly, Anderton et al. reported that EPHA2/-B4 represent the main oncogenic EPHs in BC, with other members of the EPH family also showing up- or downregulation in BC, and evidently exhibiting either tumor-promoting or tumor-suppressing capacities [21]. Notably, Zhao et al. were the first to publish a comprehensive review on EPHA2 as a promising therapeutic target in BC, and to present several available therapeutics targeting EPHA2-related pathways [22]. These targeting strategies included ephrin-A1-like antibodies or peptides, small molecular kinase inhibitors, as well as antibody–drug conjugations [22].

In the present review, we enhance the list of potential therapeutic strategies targeting EPHA2, as well as demonstrate newly developed agents targeting various members of the EPH/ephrin system in BC.

## 2. EPH/Ephrin-Targeting Therapy in BC

The EPH/ephrin system represents a promising therapeutic target for newly developed treatment agents in BC (Table 1). In terms of targeted therapy, small-molecule drugs may pass through cytomembranes to reach intracellular targets associated with the EPH/ephrin signaling cascade, while monoclonal antibodies (alone or as antibody-drug conjugates) specifically target EPHs/ephrins in their capacity as membrane-bound surface antigens. In the same context, immunoliposomes can be generated by antibody coupling to the liposomal surface, thus enabling active BC cell targeting through EPH/ephrin binding. Furthermore, adenoviral-based cancer therapy comprises the selective delivery of a therapeutic gene by adenovirus vectors to EPH/ephrin-expressing BC cells, whereas selected natural compounds target EPH/ephrin upstream positive regulators.

## 3. EPHA2-Targeting Therapeutic Agents

Dasatinib is a second-generation oral dual Bcr/Abl and Src family tyrosine kinase inhibitor (TKI) employed in the treatment of chronic myeloid leukemia and Philadelphia chromosome-positive acute lymphoblastic leukemia [37,38]. Huang et al. measured *EPHA2* expression levels after dasatinib application in sensitive BC cell lines by quantitative real-time PCR (qRT-PCR), and reported a significant, partly Src-dependent, reduction in *EPHA2* expression, phosphorylation, and kinase activity upon dasatinib treatment [23]. Interestingly, Torres-Adorno et al. combined the dasatinib application with eicosapentaenoic acid therapy, and demonstrated that combination therapy induces ATP-binding cassette sub-family A member 1 (ABCA1)-dependent cholesterol accumulation, thus increasing the plasma membrane polarity, and promoting apoptosis in TNBC cells both in vitro and in vivo [24].

Human adenovirus (HAd)-based vectors represent innovative delivery vehicles for human gene therapy [39,40,41]. Based on the assumption that EPHA2-ephrin-A1 interaction downregulates BC cell growth and survival, Noblitt et al. engineered ephrin-A1-expressing HAd-based vectors to infect MDA-MB-231 human BC cells overexpressing EPHA2. Following the infection, increased EPHA2 activation was observed, resulting in decreased BC cell viability in soft agar assays, as well as in vivo inhibition of tumor formation [31]. Similarly, Tandon et al. also employed ephrin-A1-expressing HAd-based vectors, and described in vivo ephrin-A1-EPHA2 interaction-mediated apoptosis in BC cells, as well as anticancer adaptive immune response activation, especially after combination with HAd-based vectors expressing the FMS-like tyrosine kinase receptor ligand (Flt3L) [32].

Wykosky et al. created ephrin-A1-PE38QQR, a novel cytotoxin composed of the EPHA2 ligand ephrin-A1, and PE38QQR, a mutated form of Pseudomonas aeruginosa exotoxin A, and concluded that this ephrin-A1-based cytotoxic therapy specifically acts through the EPHA2, and exhibits potent cytotoxic effects on MDA-MB-231 BC cells [33]. 

135H11 is a synthetic agent, which selectively targets EPHA2 and elicits agonistic activity only after successful dimerization or clustering. Udompholkul et al. managed to obtain multimeric 135H11 versions by biotin derivatization and streptavidin conjugation, which caused receptor clustering and internalization in MDA-MB-231 TNBC cells. Of note, fluorescently tagged streptavidin-conjugated biotinylated agents targeted TNBC cells in orthotopic mouse models as well, thus highlighting the potential use of these agents to selectively deliver chemotherapy to EPHA2-overexpressing BC [42].

Taken altogether, the newly developed targeting strategies directly target EPHA2 as a membrane receptor, inhibit its kinase activity, or mimic ephrin-A1, thus taking advantage of the EPHA2-ephrin-A1 interaction and indirectly inhibiting the EPHA2 carcinogenic functions. 

## 4. EPHA10-Targeting Therapeutic Agents

Monoclonal antibodies are laboratory-produced proteins that may specifically bind to antigens on the surface of cancer cells, thus inducing long-lasting anticancer immune responses [43]. Cha et al. generated anti-EPHA10 monoclonal antibodies, and evaluated their therapeutic efficacy in syngeneic TNBC mouse models. In vivo, the anti-EPHA10 monoclonal antibody clone #4 induced tumor regression, as well as promoted the activation of CD8+ tumor-infiltrating cytotoxic T lymphocytes (CTLs). Remarkably, the EPHA10-specific chimeric antigen receptor T lymphocytes derived from clone #4 drastically reduced TNBC growth both in vitro and in vivo, whereas the anti-EPHA10 monoclonal antibody clone #9 provoked EPHA10 internalization, thus highlighting the potential for the development of antibody–drug conjugates [25]. Analogously, Nagano et al. administered anti-EPHA10 monoclonal antibodies in a xenograft mouse model, and also noted significant in vivo TNBC growth suppression [26]. Additionally, Taki et al. crafted a dimeric bispecific antibody binding both EPHA10 and CD3, which incited an anticancer immune response by stimulating T cells to kill EPHA10-overexpressing BC cells both in vitro and in vivo [27].

These results indicate that EPHA10 represents a promising target in terms of both targeted therapy and immunotherapy, given that monoclonal antibodies not only specifically bind and interact with the membrane-bound EPHA10, but also initiate anticancer immune responses by marking EPHA10-expressing BC cells and helping the immune system recognize and destroy them.

## 5. EPHB4-Targeting Therapeutic Agents

Sanguinarine is a natural benzophenanthridine alkaloid that seemingly regulates apoptotic signaling pathways, and has been proposed as a potential treatment agent for chronic human diseases [44]. A Chinese study group investigated the effect of sanguinarine and reported effective downregulation of hypoxia-inducible factor-1α (HIF-1α) and hypoxia-induced EPHB4, and consequent signal transducer and activator of transcription-3 (STAT3) activation in BC [34]. In this context, sanguinarine alternatively targets hypoxia-induced upstream positive regulators, in order to downregulate the EPHB4 expression in BC cells. 

## 6. Ephrin-A2-Targeting Therapeutic Agents

Immunoliposomes are immunoglobulins coupled to the liposomal surface that bind to tumor cell-specific receptors and enable active tissue targeting [45]. Huang et al. generated the ephrin-A2 targeted taxane liposomal prodrug 2, the application of which resulted in profound tumor regression in the TNBC xenograft models MDA-MB-436 and SUM149 [30]. Notably, the immunoliposome displayed equilibrium dissociation constantly toward the extracellular ephrin-A2 receptor domain [30]. 

## 7. Ephrin-A4-Targeting Therapeutic Agents

PF-06647263 is an antibody–drug conjugate composed of a humanized anti-ephrin-A4 monoclonal antibody conjugated to the DNA-damaging agent calicheamicin. Damelin et al. were the first to evaluate the in vivo efficacy of PF-06647263 in patient-derived TNBC xenograft models, and to report constant TNBC regression, especially in non-claudin low TNBC tumors [28]. Garrido-Laguna et al., then, performed the first-in-human, phase I study of PF-06647263 in women with pretreated, metastatic TNBC, and concluded that the weekly administration of PF-06647263 at the recommended dose of 0.015 mg/kg could be well tolerated. Nevertheless, despite evident antitumor activity in heavily pretreated TNBC, study enrollment was terminated, given the inadequate response to PF-06647263 exposure [29]. Altogether, ephrin-A4 can serve as a target for antibody–drug conjugation.

## 8. Ephrin-B2-Targeting Therapeutic Agents

Barneh et al. stimulated MDA-MB-231 BC cells by diverse concentrations of pre-clustered ephrin-B2-Fc, and underlined a dose-dependent, EPHB4-mediated TNBC growth inhibition after six days, with the cells being in a post-confluent state [46]. Similarly, Noren et al. described in vivo EPHB4-mediated, Abl-Crk-dependent BC cell growth and motility, as well as invasion inhibition upon ephrin-B2-Fc treatment [47].

Berberine is a plant-extracted isoquinoline alkaloid with multiple pharmacologic activities in various disorders [48]. Ma et al. investigated the effect of berberine on BC cell growth and migration, and reported downregulated matrix metalloproteinase (MMP)-2/-9 expression, diminished vascular endothelial growth factor receptor 2 (VEGFR2) phosphorylation, as well as ephrin-B2 and its PDZ binding proteins decrease, leading to reduced ZR-75-30 BC cell proliferation and migration [35]. 

All in all, the ephrin-B2-Fc treatment seems to exclusively exhibit EPHB4-mediated anti-BC effects, whereas berberine inhibits BC cell growth and migration by selective ephrin-B2 targeting.

## 9. Multiple EPH/ephrin-Targeting Therapeutic Agents

Artesunate is an artemisinin derivative representing a potent antimalarial agent [49]. Zadeh et al. grew MCF7 and MDA-MB-231 BC cells in the presence of different artesunate concentrations, and found markedly increased *EPHA8*, *EPHA10*, *EPHB6,* and *ephrin-A2* levels in MCF7 cells. In MDA-MB-231 cells, *EPHA3* and *EPHA10* levels were significantly elevated, whereas *EPHA7* and *ephrin-A3* were downregulated [36]. Consequently, artesunate differentially regulates *EPH*/*ephrin* expression depending on the BC cell line. 

Table 2 summarizes the effects of different therapeutic agents on the EPH/ephrin system in BC.

**Figure 1:**
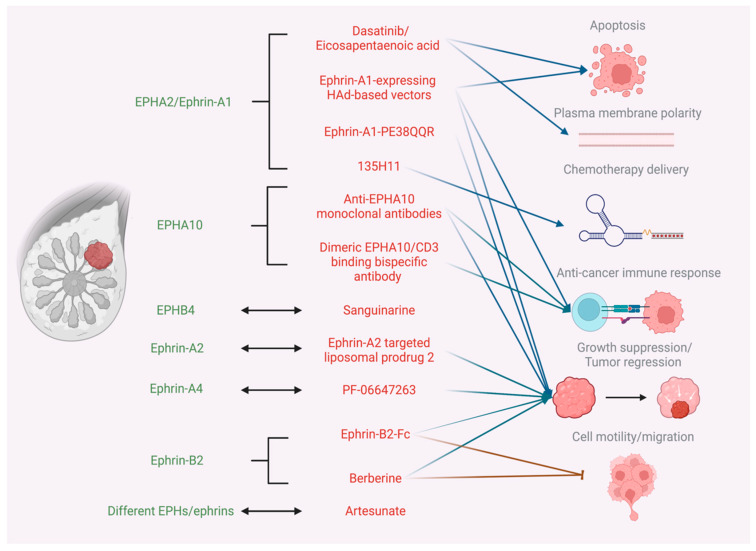
Mechanisms of action of different therapeutic agents targeting the EPH/ephrin system in BC. Created with BioRender.com.

Figure 1 depicts the mechanisms of action of different EPH/ephrin-targeting therapeutic agents in BC.

## 10. Conclusions

Due to its implication in various cancer types, the EPH/ephrin system has long represented a feasible and most promising target for anticancer treatment [50]. To date, a growing number of EPH/ephrin-targeting therapeutic agents is in advanced preclinical development, or has even entered phase I/II clinical investigation [51,52], thus encouraging international scientific groups to further develop novel agents targeting this system in different cancer entities. Especially in terms of BC, the generation of efficient anticancer drugs is of utmost importance, given that the 5-year survival rate for women diagnosed with metastatic BC in a distant Surveillance, Epidemiology, and End Results (SEER) stage amounts to 29% [53]. 

The current review summarizes the results of original research articles extensively studying the mechanisms of action of newly established agents, ranging from TKIs or HAd-based vectors to immunotherapeutics and phytopharmaceuticals, and targeting diverse members of the EPH/ephrin family in BC. Novel studies on the use of therapeutics targeting EPHA2-related pathways were identified, hence completing the list of available EPHA2-targeted therapies in BC [22]. With the list now containing a total of 19 relevant preclinical studies, EPHA2 is, undoubtedly, the most studied EPH/ephrin target in BC treatment, the targeting of which mainly results in cell death induction, tumor growth inhibition, as well as anticancer immune system response. Nonetheless, none of the described agents has, to date, been tested in clinical trials, which would provide essential information on the feasibility of their introduction in the clinical routine. Furthermore, targeting of EPHA10, EPHB4, ephrin-A2, and ephrin-A4, as well as ephrin-B2 in BC cells or xenograft models was associated with tumor regression, anticancer T-cell activation, and impaired cell motility. Of note, Garrido-Laguna et al. first performed a phase I clinical trial, and concluded that PF-06647263 exerts antitumor effects in pretreated, metastatic TNBC [29]. In summary, the generation of EPH/ephrin-targeting therapeutic agents lays the foundation for the establishment of a novel, efficient therapeutic regimen, that may endorse, or even replace standard chemotherapeutic approaches, and provide better treatment options for especially aggressive BC subtypes. Ideally, clinical trials in large patient collectives need to be conducted, with a view to verifying the clinical utility and safety of the agents targeting members of the EPH/ephrin system in BC, investigating eventual adverse side effects following their administration to patients, as well as determining their efficacy depending on EPH/ephrin expression by BC cells.

## Figures and Tables

**Table 1:** Main available therapeutics targeting the EPH/ephrin system in BC.

Targeting Strategy	Mechanism of Action	References
Small molecule drugs	Tyrosine kinase inhibitors	[23,24]
Monoclonal antibodies	Anti-EPH/ephrin monoclonal antibodies, Antibody-drug conjugations	[25,26,27,28,29]
Immunoliposomes	Antibodies coupled to the liposomal surface	[30]
Adenoviral-based cancer therapy	Human adenovirus-based vectors	[31,32]
Natural compounds	Cytotoxins, Alkaloids, Artemisinin	[33,34,35,36]

**Table 2:** Effects of different therapeutic agents on the EPH/ephrin system in BC.

Therapeutic Agent	Targeted EPH/Ephrin	Mechanism of Action	References
Dasatinib/ Eicosapentaenoic acid	EPHA2	• Reduction in *EPHA2* expression, phosphorylation, and kinase activity • Plasma membrane polarity increase through ABCA1-dependent cholesterol accumulation • Apoptosis induction	[23,24]
Ephrin-A1-expressing HAd-based vectors	Ephrin-A1/EPHA2	• EPHA2 upregulation • Decreased BC cell viability • Inhibition of tumor formation • Apoptosis induction • Anticancer adaptive immune response activation	[31,32]
Ephrin-A1-PE38QQR	Ephrin-A1/EPHA2	• Cytotoxic effects on BC cells	[33]
135H11	EPHA2	• Agonistic activity only after successful dimerization or clustering • Selective chemotherapy delivery	[42]
Anti-EPHA10 monoclonal antibodies	EPHA10	• Tumor regression induction • BC growth suppression • CD8+ tumor-infiltrating CTL activation • EPHA10 internalization	[25,26]
Dimeric EPHA10/CD3 binding bispecific antibody	EPHA10	• Anticancer T-cell stimulation	[27]
Sanguinarine	EPHB4	• Down regulation of hypoxia- induced EPHB4 • STAT3 activation	[34]
Ephrin-A2 targeted liposomal prodrug 2	Ephrin-A2	• BC tumor regression	[30]
PF-06647263	Ephrin-A4	• BC tumor regression	[28,29]
Ephrin-B2-Fc	Ephrin-B2/EPHB4	• Dose- dependent, EPHB4- mediated BC growth inhibition • Inhibition of EPHB4- mediated, Abl-Crk -dependent BC cell growth, motility, and invasion	[46,47]
Berberine	Ephrin-B2	• Reduced ZR-75-30 BC cell proliferation and migration	[35]
Artesunate	EPHA3, EPHA7, EPHA8, EPHA10, EPHB6, ephrin-A2, ephrin-A3	• *EPHA8*, *EPHA10*, *EPHB6* and *ephrin-A2* upregulation in MCF7 cells • *EPHA3* and *EPHA10* upregulation in MDA-MB-231 cells • *EPHA7* and *ephrin-A3* down regulation in MDA-MB-231 cells	[36]

## Data Availability

Not applicable.
